# *catena*-Poly[[[bis­[aqua­nickel(II)]bis­(μ-pyridine-2,6-dicarboxyl­ato *N*-oxide)]-μ-1,2-di-4-pyridylethane] tetra­hydrate]

**DOI:** 10.1107/S1600536808031619

**Published:** 2008-10-04

**Authors:** Ming-Hua Yang

**Affiliations:** a Department of Chemistry, Lishui University, Lishui 323000, People’s Republic of China

## Abstract

In the title compound, {[Ni_2_(C_7_H_3_NO_5_)_2_(C_12_H_12_N_2_)(H_2_O)_2_]·4H_2_O}_*n*_, two Ni^II^ ions, two tridentate pyridine-2,6-dicarboxyl­ate *N*-oxide ligands and two coordinated water mol­ecules form centrosymmetric dinuclear units, which are further bridged by centrosymmetric 1,2-di-4-pyridylethane ligands into polymeric chains along [210]. Each Ni^II^ ion has a distorted square-pyramidal environment, with the basal plane formed by three O [Ni—O = 1.9290 (16)–1.9588 (10) Å] and one N [Ni—N = 1.9828 (18) Å] atoms and the apical position occupied by the water mol­ecule [Ni—O = 2.2643 (11) Å]. The water mol­ecules are involved in the formation of O—H⋯O hydrogen bonds.

## Related literature

For related literature, see: Laine *et al.* (1995*a* ▶,*b* ▶); Lin *et al.* (2006 ▶); Nathan *et al.* (1985 ▶). For a related structure, see: Wen *et al.* (2005 ▶).
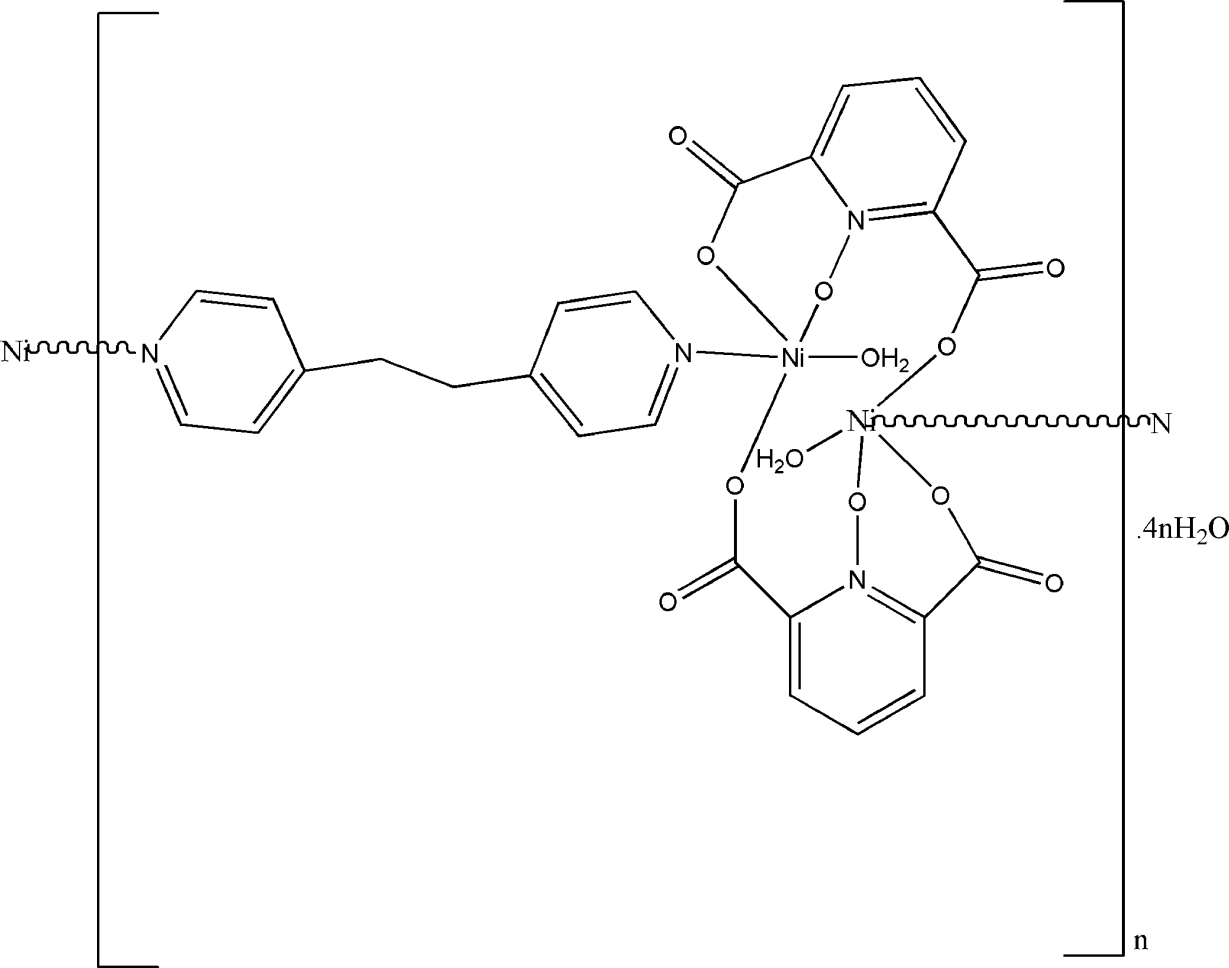


## Experimental

### 

#### Crystal data

[Ni_2_(C_7_H_3_NO_5_)_2_(C_12_H_12_N_2_)(H_2_O)_2_]·4H_2_O
*M* *_r_* = 771.96
Triclinic, 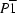

*a* = 8.2803 (16) Å
*b* = 10.3542 (15) Å
*c* = 11.1326 (16) Å
α = 113.727 (2)°
β = 104.282 (2)°
γ = 100.255 (2)°
*V* = 804.4 (2) Å^3^
*Z* = 1
Mo *K*α radiation
μ = 1.25 mm^−1^
*T* = 298 (2) K
0.25 × 0.19 × 0.16 mm

#### Data collection

Bruker APEXII area-detector diffractometer
Absorption correction: multi-scan (*SADABS*; Sheldrick, 1996 ▶) *T*_min_ = 0.745, *T*_max_ = 0.825
4146 measured reflections
2850 independent reflections
2180 reflections with *I* > 2σ(*I*)
*R*_int_ = 0.033

#### Refinement

*R*[*F* ^2^ > 2σ(*F* ^2^)] = 0.028
*wR*(*F* ^2^) = 0.063
*S* = 0.83
2850 reflections
199 parameters
H-atom parameters constrained
Δρ_max_ = 0.35 e Å^−3^
Δρ_min_ = −0.29 e Å^−3^

### 

Data collection: *APEX2* (Bruker, 2004 ▶); cell refinement: *APEX2*; data reduction: *APEX2*; program(s) used to solve structure: *SHELXS97* (Sheldrick, 2008 ▶); program(s) used to refine structure: *SHELXL97* (Sheldrick, 2008 ▶); molecular graphics: *ORTEPIII* (Burnett & Johnson, 1996 ▶), *ORTEP-3 for Windows* (Farrugia, 1997 ▶) and *PLATON* (Spek, 2003 ▶); software used to prepare material for publication: *SHELXL97*.

## Supplementary Material

Crystal structure: contains datablocks I, global. DOI: 10.1107/S1600536808031619/cv2458sup1.cif

Structure factors: contains datablocks I. DOI: 10.1107/S1600536808031619/cv2458Isup2.hkl

Additional supplementary materials:  crystallographic information; 3D view; checkCIF report

## Figures and Tables

**Table 1:** Hydrogen-bond geometry (Å, °)

*D*—H⋯*A*	*D*—H	H⋯*A*	*D*⋯*A*	*D*—H⋯*A*
O1*W*—H1*W*⋯O3*W*^i^	0.85	1.99	2.8144 (15)	162
O1*W*—H2*W*⋯O2^i^	0.86	1.98	2.824 (2)	168
O3*W*—H6*W*⋯O5^i^	0.85	1.94	2.7791 (13)	170
O3*W*—H5*W*⋯O2*W*	0.86	2.44	2.8527 (13)	110
O2*W*—H3*W*⋯O2	0.84	2.15	2.976 (2)	167
O2*W*—H4*W*⋯O3*W*^ii^	0.84	1.97	2.7985 (14)	169

Symmetry codes: (i) 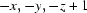; (ii) 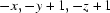.

**Fig. 1.**
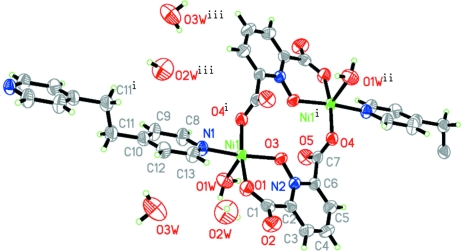
A portion of the crystal structure of (I) showing the atomic numbering scheme and 40% probability displacement ellipsoids [symmetry codes: (i) -x, -y, 2 - z; (ii) -x, -y, 1 - z' (iii) -x, 1 - y, 1 - z].

## supplementary crystallographic information

### Comment

The complexation of metal ions by dicarboxylate acid (pyridine-2,6-dicarboxylic acid) has been extensively studied (Laine *et al.*, 1995*a,b*). Owing to the unique ability of the ligand to form stable chelates with various coordination modes and its biological activity, many crystal structures have been determined. Pyridine-2,6-dicarboxylic acid N-oxide (pydco), has limited steric hindrance and weak stacking interactions and can offer possibilities to form complicated coordination polymers through polycarboxylate ligands. However, the coordination chemistry and structural properties of metal polymers containing pydco ligands have seldom been documented to date (Nathan *et al.*, 1985; Lin *et al.*, 2006; Wen *et al.*, 2005). In this paper, we report the synthesis and crystal structure of the title compound, (I).

In (I) (Fig. 1), each Ni^II^ atom is coordinated by three oxygen atoms from the carboxylato groups and one N-oxide entity from two pydco anions and one N atom from bridging 1,2-di-4-pyridylethane (bpa) ligand to form the basal plane, and further it coordinated by one apical oxygen atom from one water molecule to form a quasi-square pyramidal environment. Each carboxylato group is coordinated to the Ni atom in a monodentate fashion and the two carboxyl groups are out of coplanarity with the correspondingly linking pyridine rings, with the dihedral angles between them being *ca* 46° and 39°, respectively. They are very different from those in the free H_2_pydco, in which the carboxyl groups are found to be essentially coplanar with the pyridine rings. Owing to the monodentate coordination modes of carboxylate groups, a binuclear [Ni_2_(pydco)_2_] unit was formed. Finally, bpa ligands connect the dimeric units into polymeric zigzag chain.

The crystalline water molecules contribute to intermolecular O—H···O hydrogen bonds (Table 1), which stabilize the crystal packing.

### Experimental

Ni(AC)_2_ (25 mg, 0.07 mmol), H_2_pydco (31 mg, 0.15 mmol), bpa (19 mg, 0.09 mmol) were added in a solvent of acetonitrile, the mixture was heated for two hours under reflux. during the process stirring and influx were required. The resultant was kept at room temperature for six weeks, when single crystals were obtained.

### Refinement

C-bound H atoms were geometrically positioned (C—H 0.93-0.97 Å). The O-bound H atoms were located on a Fourier difference map with O—H 0.84-0.86 °. All H atoms were refined as riding, with *U*_iso_(H) = 1.2-1.5*U*_eq_ of the parent atom.

### Crystal data

**Table:**

[Ni_2_(C_7_H_3_NO_5_)_2_(C_12_H_12_N_2_)(H_2_O)_2_]·4H_2_O	*Z* = 1
*M**_r_* = 771.96	*F*(000) = 398
Triclinic, *P*1	*D*_x_ = 1.594 Mg m^−^^3^
Hall symbol: -P 1	Mo *K*α radiation, λ = 0.71073 Å
*a* = 8.2803 (16) Å	Cell parameters from 2850 reflections
*b* = 10.3542 (15) Å	θ = 2.1–25.2°
*c* = 11.1326 (16) Å	µ = 1.25 mm^−^^1^
α = 113.727 (2)°	*T* = 298 K
β = 104.282 (2)°	Block, green
γ = 100.255 (2)°	0.25 × 0.19 × 0.16 mm
*V* = 804.4 (2) Å^3^	

### Data collection

**Table:**

Bruker APEXII area-detector diffractometer	2850 independent reflections
Radiation source: fine-focus sealed tube	2180 reflections with *I* > 2σ(*I*)
graphite	*R*_int_ = 0.033
φ and ω scans	θ_max_ = 25.2°, θ_min_ = 2.1°
Absorption correction: multi-scan (SADABS; Sheldrick, 1996)	*h* = −9→9
*T*_min_ = 0.745, *T*_max_ = 0.825	*k* = −12→12
4146 measured reflections	*l* = −13→12

### Refinement

**Table:**

Refinement on *F*^2^	Primary atom site location: structure-invariant direct methods
Least-squares matrix: full	Secondary atom site location: difference Fourier map
*R*[*F*^2^ > 2σ(*F*^2^)] = 0.028	Hydrogen site location: inferred from neighbouring sites
*wR*(*F*^2^) = 0.063	H-atom parameters constrained
*S* = 0.83	*w* = 1/[σ^2^(*F*_o_^2^) + (0.0269*P*)^2^ + 0.19*P*] where *P* = (*F*_o_^2^ + 2*F*_c_^2^)/3
2850 reflections	(Δ/σ)_max_ < 0.001
199 parameters	Δρ_max_ = 0.35 e Å^−^^3^
0 restraints	Δρ_min_ = −0.29 e Å^−^^3^

### Special details

**Table:**

Geometry. All e.s.d.'s (except the e.s.d. in the dihedral angle between two l.s. planes) are estimated using the full covariance matrix. The cell e.s.d.'s are taken into account individually in the estimation of e.s.d.'s in distances, angles and torsion angles; correlations between e.s.d.'s in cell parameters are only used when they are defined by crystal symmetry. An approximate (isotropic) treatment of cell e.s.d.'s is used for estimating e.s.d.'s involving l.s. planes.
Refinement. Refinement of *F*^2^ against ALL reflections. The weighted *R*-factor *wR* and goodness of fit *S* are based on *F*^2^, conventional *R*-factors *R* are based on *F*, with *F* set to zero for negative *F*^2^. The threshold expression of *F*^2^ > σ(*F*^2^) is used only for calculating *R*-factors(gt) *etc*. and is not relevant to the choice of reflections for refinement. *R*-factors based on *F*^2^ are statistically about twice as large as those based on *F*, and *R*- factors based on ALL data will be even larger.

### Fractional atomic coordinates and isotropic or equivalent isotropic displacement parameters (Å^2^)

**Table:**

	*x*	*y*	*z*	*U*_iso_*/*U*_eq_	
Ni1	0.19610 (4)	0.10188 (3)	0.85792 (3)	0.03124 (11)	
O1	0.0912 (2)	0.1451 (2)	0.70832 (19)	0.0473 (5)	
O2	−0.1257 (2)	0.1548 (2)	0.5550 (2)	0.0600 (6)	
O3	−0.03449 (19)	0.0177 (2)	0.85274 (17)	0.0433 (4)	
N1	0.4335 (2)	0.2138 (2)	0.8837 (2)	0.0376 (5)	
N2	−0.1753 (2)	−0.0555 (2)	0.7358 (2)	0.0354 (5)	
C1	−0.0673 (3)	0.1028 (3)	0.6318 (3)	0.0401 (6)	
C2	−0.2011 (3)	−0.0227 (3)	0.6272 (3)	0.0368 (6)	
C3	−0.3544 (3)	−0.1043 (3)	0.5128 (3)	0.0489 (7)	
H3	−0.3736	−0.0847	0.4369	0.059*	
C4	−0.4795 (4)	−0.2144 (3)	0.5092 (3)	0.0570 (8)	
H4	−0.5824	−0.2686	0.4315	0.068*	
C5	−0.4508 (3)	−0.2433 (3)	0.6215 (3)	0.0492 (7)	
H5	−0.5343	−0.3172	0.6205	0.059*	
C6	−0.2980 (3)	−0.1624 (3)	0.7350 (3)	0.0366 (6)	
C8	0.5711 (3)	0.1727 (3)	0.9306 (3)	0.0490 (7)	
H8	0.5514	0.0953	0.9529	0.059*	
C9	0.7394 (3)	0.2397 (3)	0.9470 (3)	0.0507 (7)	
H9	0.8307	0.2077	0.9798	0.061*	
C10	0.7726 (3)	0.3553 (3)	0.9144 (3)	0.0436 (6)	
C11	0.9551 (3)	0.4316 (3)	0.9304 (3)	0.0518 (7)	
H11A	1.0234	0.3635	0.9215	0.062*	
H11B	0.9488	0.4587	0.8559	0.062*	
C12	0.6307 (3)	0.3995 (3)	0.8690 (3)	0.0479 (7)	
H12	0.6474	0.4782	0.8482	0.058*	
C13	0.46548 (10)	0.32671 (9)	0.85477 (8)	0.0442 (7)	
H13	0.3722	0.3577	0.8236	0.053*	
O1W	0.22201 (10)	−0.11885 (9)	0.72089 (8)	0.0584 (5)	
H1W	0.1610	−0.1939	0.7212	0.088*	
H2W	0.2045	−0.1367	0.6357	0.088*	
O2W	0.15515 (10)	0.41900 (9)	0.61420 (8)	0.0944 (8)	
H3W	0.0828	0.3364	0.5887	0.142*	
H4W	0.1122	0.489	0.6354	0.142*	
O3W	0.03080 (10)	0.37588 (9)	0.33371 (8)	0.0875 (7)	
H6W	0.0820	0.3592	0.2741	0.131*	
H5W	0.1116	0.4510	0.4044	0.131*	
C7	−0.25478 (10)	−0.18425 (9)	0.86469 (8)	0.0374 (6)	
O4	−0.28538 (10)	−0.09359 (9)	0.96606 (8)	0.0401 (4)	
O5	−0.19822 (10)	−0.28825 (9)	0.85957 (8)	0.0540 (5)	

### Atomic displacement parameters (Å^2^)

**Table:**

	*U*^11^	*U*^22^	*U*^33^	*U*^12^	*U*^13^	*U*^23^
Ni1	0.02697 (17)	0.0386 (2)	0.03728 (19)	0.00914 (13)	0.01384 (14)	0.02526 (16)
O1	0.0408 (10)	0.0605 (12)	0.0545 (12)	0.0133 (9)	0.0172 (9)	0.0406 (11)
O2	0.0654 (13)	0.0709 (14)	0.0579 (13)	0.0196 (11)	0.0122 (10)	0.0495 (12)
O3	0.0302 (9)	0.0632 (12)	0.0351 (10)	0.0041 (8)	0.0070 (8)	0.0286 (9)
N1	0.0335 (11)	0.0434 (13)	0.0472 (13)	0.0122 (10)	0.0175 (10)	0.0297 (11)
N2	0.0296 (11)	0.0454 (13)	0.0341 (12)	0.0114 (10)	0.0107 (10)	0.0218 (11)
C1	0.0484 (16)	0.0430 (16)	0.0361 (15)	0.0176 (13)	0.0183 (13)	0.0219 (13)
C2	0.0388 (14)	0.0456 (16)	0.0335 (14)	0.0180 (12)	0.0152 (12)	0.0221 (13)
C3	0.0471 (16)	0.0630 (19)	0.0386 (16)	0.0170 (15)	0.0102 (14)	0.0285 (15)
C4	0.0403 (16)	0.073 (2)	0.0413 (17)	0.0064 (15)	0.0009 (14)	0.0243 (16)
C5	0.0403 (15)	0.0570 (19)	0.0440 (17)	0.0067 (13)	0.0102 (13)	0.0241 (15)
C6	0.0319 (13)	0.0431 (16)	0.0374 (15)	0.0096 (12)	0.0129 (12)	0.0219 (13)
C8	0.0419 (15)	0.0528 (18)	0.070 (2)	0.0182 (13)	0.0246 (15)	0.0413 (16)
C9	0.0376 (15)	0.0531 (18)	0.070 (2)	0.0172 (13)	0.0220 (14)	0.0340 (16)
C10	0.0384 (14)	0.0430 (16)	0.0456 (16)	0.0072 (12)	0.0191 (13)	0.0172 (14)
C11	0.0420 (16)	0.0497 (18)	0.0549 (18)	0.0048 (13)	0.0224 (14)	0.0172 (14)
C12	0.0497 (16)	0.0403 (16)	0.0560 (18)	0.0061 (13)	0.0202 (14)	0.0270 (15)
C13	0.0400 (15)	0.0445 (16)	0.0553 (17)	0.0127 (13)	0.0167 (13)	0.0305 (15)
O1W	0.0732 (13)	0.0580 (13)	0.0531 (12)	0.0221 (11)	0.0286 (11)	0.0301 (11)
O2W	0.1038 (19)	0.0682 (16)	0.113 (2)	0.0268 (14)	0.0349 (16)	0.0454 (15)
O3W	0.127 (2)	0.0742 (16)	0.0925 (17)	0.0440 (15)	0.0679 (16)	0.0463 (14)
C7	0.0256 (13)	0.0449 (17)	0.0418 (16)	0.0028 (12)	0.0102 (12)	0.0249 (14)
O4	0.0350 (9)	0.0520 (11)	0.0420 (10)	0.0147 (8)	0.0165 (8)	0.0278 (9)
O5	0.0675 (13)	0.0502 (12)	0.0608 (13)	0.0252 (10)	0.0269 (11)	0.0360 (11)

### Geometric parameters (Å, °)

**Table:**

Ni1—O3	1.9290 (16)	C8—C9	1.373 (3)
Ni1—O1	1.9373 (16)	C8—H8	0.9300
Ni1—O4^i^	1.9588 (10)	C9—C10	1.386 (3)
Ni1—N1	1.9828 (18)	C9—H9	0.9300
Ni1—O1W	2.2643 (11)	C10—C12	1.390 (3)
O1—C1	1.258 (3)	C10—C11	1.506 (3)
O2—C1	1.230 (3)	C11—C11^ii^	1.501 (5)
O3—N2	1.331 (2)	C11—H11A	0.9700
N1—C13	1.3332 (19)	C11—H11B	0.9700
N1—C8	1.345 (3)	C12—C13	1.376 (3)
N2—C6	1.358 (3)	C12—H12	0.9300
N2—C2	1.361 (3)	C13—H13	0.9300
C1—C2	1.525 (3)	O1W—H1W	0.85
C2—C3	1.379 (3)	O1W—H2W	0.86
C3—C4	1.377 (4)	O2W—H3W	0.84
C3—H3	0.9300	O2W—H4W	0.84
C4—C5	1.375 (3)	O3W—H6W	0.85
C4—H4	0.9300	O3W—H5W	0.86
C5—C6	1.371 (3)	C7—O5	1.2351 (14)
C5—H5	0.9300	C7—O4	1.2618 (12)
C6—C7	1.517 (3)	O4—Ni1^i^	1.9588 (10)
			
O3—Ni1—O1	89.83 (7)	N2—C6—C5	120.3 (2)
O3—Ni1—O4^i^	86.14 (5)	N2—C6—C7	116.2 (2)
O1—Ni1—O4^i^	167.38 (6)	C5—C6—C7	123.6 (3)
O3—Ni1—N1	172.23 (8)	N1—C8—C9	123.1 (2)
O1—Ni1—N1	90.76 (7)	N1—C8—H8	118.5
O4^i^—Ni1—N1	91.65 (6)	C9—C8—H8	118.5
O3—Ni1—O1W	94.95 (6)	C8—C9—C10	119.7 (2)
O1—Ni1—O1W	97.00 (6)	C8—C9—H9	120.2
O4^i^—Ni1—O1W	95.30 (6)	C10—C9—H9	120.2
N1—Ni1—O1W	92.67 (6)	C9—C10—C12	117.0 (2)
C1—O1—Ni1	129.11 (16)	C9—C10—C11	121.5 (2)
N2—O3—Ni1	123.58 (13)	C12—C10—C11	121.5 (2)
C13—N1—C8	117.39 (17)	C11^ii^—C11—C10	111.5 (3)
C13—N1—Ni1	123.82 (12)	C11^ii^—C11—H11A	109.3
C8—N1—Ni1	118.78 (15)	C10—C11—H11A	109.3
O3—N2—C6	114.80 (18)	C11^ii^—C11—H11B	109.3
O3—N2—C2	123.49 (19)	C10—C11—H11B	109.3
C6—N2—C2	121.6 (2)	H11A—C11—H11B	108.0
O2—C1—O1	124.3 (2)	C13—C12—C10	120.0 (2)
O2—C1—C2	115.3 (2)	C13—C12—H12	120.0
O1—C1—C2	120.5 (2)	C10—C12—H12	120.0
N2—C2—C3	118.2 (2)	N1—C13—C12	122.78 (15)
N2—C2—C1	121.5 (2)	N1—C13—H13	118.6
C3—C2—C1	120.3 (2)	C12—C13—H13	118.6
C4—C3—C2	121.1 (2)	Ni1—O1W—H1W	115.3
C4—C3—H3	119.5	Ni1—O1W—H2W	114.2
C2—C3—H3	119.5	H1W—O1W—H2W	108.3
C5—C4—C3	119.4 (3)	H3W—O2W—H4W	113.1
C5—C4—H4	120.3	H6W—O3W—H5W	99.6
C3—C4—H4	120.3	O5—C7—O4	127.3 (1)
C6—C5—C4	119.5 (2)	O5—C7—C6	117.9 (2)
C6—C5—H5	120.3	O4—C7—C6	114.9 (2)
C4—C5—H5	120.3	C7—O4—Ni1^i^	114.7 (1)

Symmetry codes: (i) −*x*, −*y*, −*z*+2; (ii) −*x*+2, −*y*+1, −*z*+2.

### Hydrogen-bond geometry (Å, °)

**Table:**

*D*—H···*A*	*D*—H	H···*A*	*D*···*A*	*D*—H···*A*
O1W—H1W···O3W^iii^	0.85	1.99	2.8144 (15)	162
O1W—H2W···O2^iii^	0.86	1.98	2.824 (2)	168
O3W—H6W···O5^iii^	0.85	1.94	2.7791 (13)	170
O3W—H5W···O2W	0.86	2.44	2.8527 (13)	110
O2W—H3W···O2	0.84	2.15	2.976 (2)	167
O2W—H4W···O3W^iv^	0.84	1.97	2.7985 (14)	169

Symmetry codes: (iii) −*x*, −*y*, −*z*+1; (iv) −*x*, −*y*+1, −*z*+1.
